# Successful rescue of the amputated glans penis placed as a composite graft and the application of continuous papaverine: A case report

**DOI:** 10.1016/j.ijscr.2025.112094

**Published:** 2025-10-24

**Authors:** Serdar Nazif Nasır

**Affiliations:** a Department of Plastic, Reconstructive and Aesthetic Surgery, Faculty of Medicine, Hacettepe University, Ankara, Türkiye

**Keywords:** Glans penis amputation, Circumcision complications, Composite graft, Papaverine, Penis reconstruction, Case report

## Abstract

**Introduction and importance:**

Penile amputation is an uncommon condition reported globally as isolated cases or in small series. The leading cause of penile amputation is psychiatric disorders and self-amputation of external genitalia with hallucinations and delusions stemming from substance abuse. The chance of applying microsurgery is higher since self-mutilation in these cases occurs more frequently near the proximal part of the penis. In contrast, penile amputations caused by circumcision typically occur at the glans level, where microsurgical repair is usually not feasible. Therefore, utilizing a composite graft remains the only option.

**Case presentation:**

We present a papaverine application protocol designed to enhance the neovascularization of the composite glans penis graft and maintain graft viability at the highest possible level.

**Clinical discussion:**

Distal penile amputations remain surgically challenging. Composite grafting is reported for glans amputations; however, success rates are limited, and necrosis is a common complication. In our case, the use of topical papaverine may have supported neovascularization and graft survival. Additionally, penile amputation cases due to circumcision in the English and non-English literature are reviewed.

**Conclusion:**

Composite grafting is the only feasible option for distal penile amputations. Topical papaverine can increase neovascularization, thereby minimizing graft loss, especially in thick tissues such as composite grafts, although larger clinical studies are needed to validate its efficacy.

## Highlights

• This is the first report describing the use of continuous papaverine to support the survival of a glans penis composite graft.
• In circumcision-related amputations at the glans level, microsurgery is not possible; composite grafting remains the only reconstructive option. Some reports in the literature describe ‘microsurgical repair,’ but in fact these were composite grafts without true vascular anastomosis.
• Continuous papaverine may improve graft acceptance and could be relevant for other large or thick grafts.

## 1 Introduction

Penile amputation trauma is a rare emergency and has various causes, including self-mutilation secondary to psychosis, domestic violence, burns or industrial accidents, and injuries inflicted by improper circumcision in men. This trauma can result in complications such as urination disorders, erectile dysfunction, and sensory problems [1,2]. The treatment involves maintaining the function and sensation of the penis as well as the capacity of urination [3]. We report the method of penis repair using a composite tissue graft and papaverine due to glans penis amputation.

This case has been reported in line with the SCARE criteria. [Kerwan A, Al-Jabir A, Mathew G, Sohrabi C, Rashid R, Franchi T, Nicola M, Agha M, Agha RA. Revised Surgical CAse REport (SCARE) guideline: An update for the age of Artificial Intelligence. Premier Journal of Science 2025:10; 100079.]

## 2 Case

**Fig. 1:**
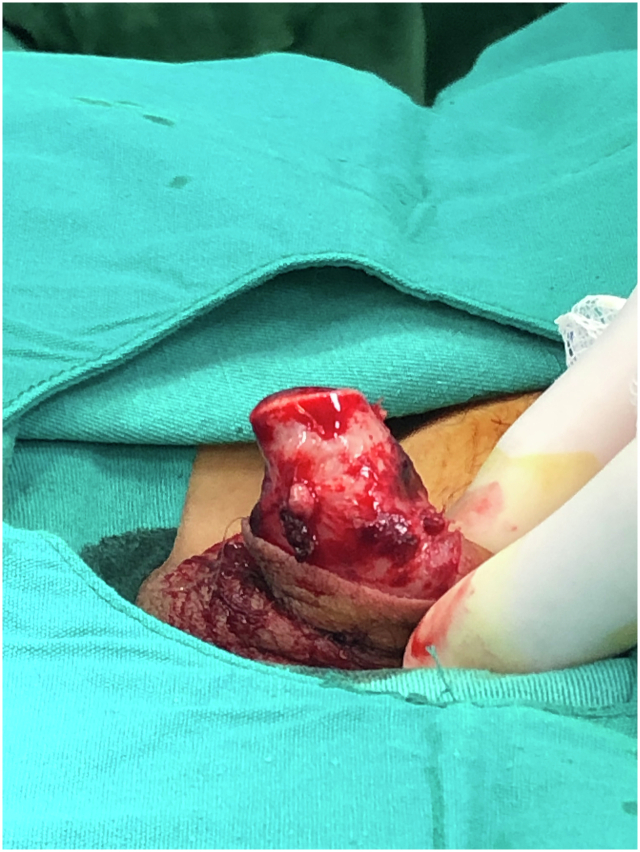
Penis stump depends on iatrogenic amputation during circumcision.

**Fig. 2:**
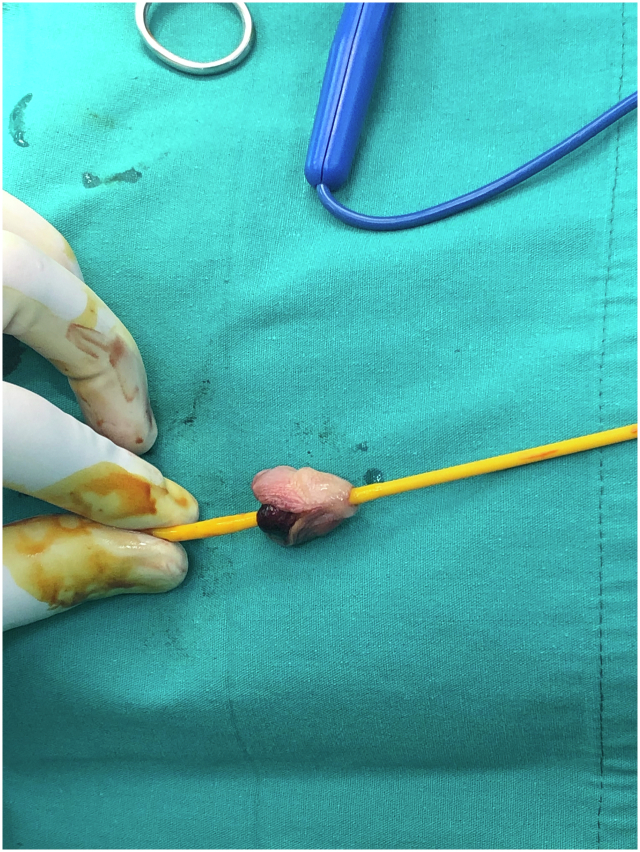
Foley catheter was inserted urethra of the composite glans penis graft.

**Fig. 3:**
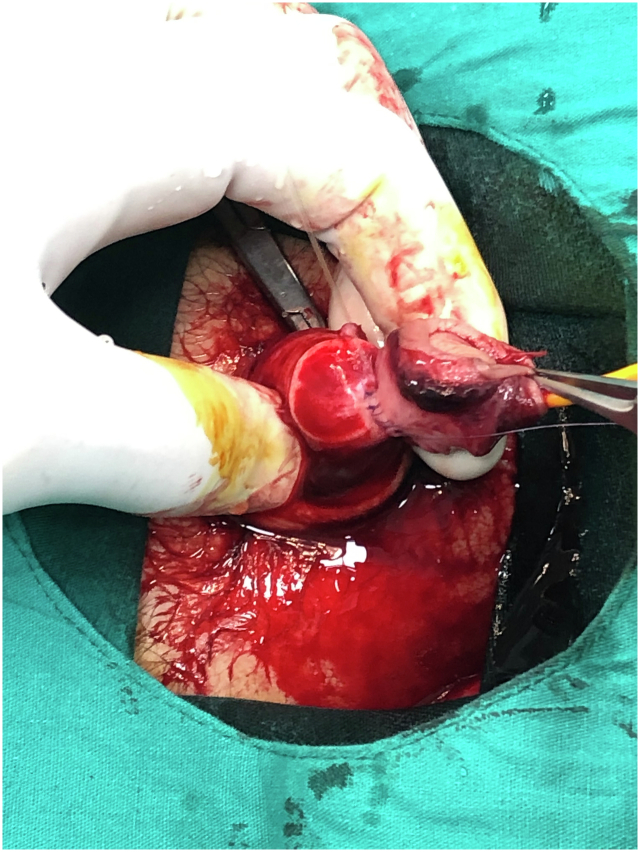
Completed urethra repair between the stump and the composite glans penis graft.

**Fig. 4:**
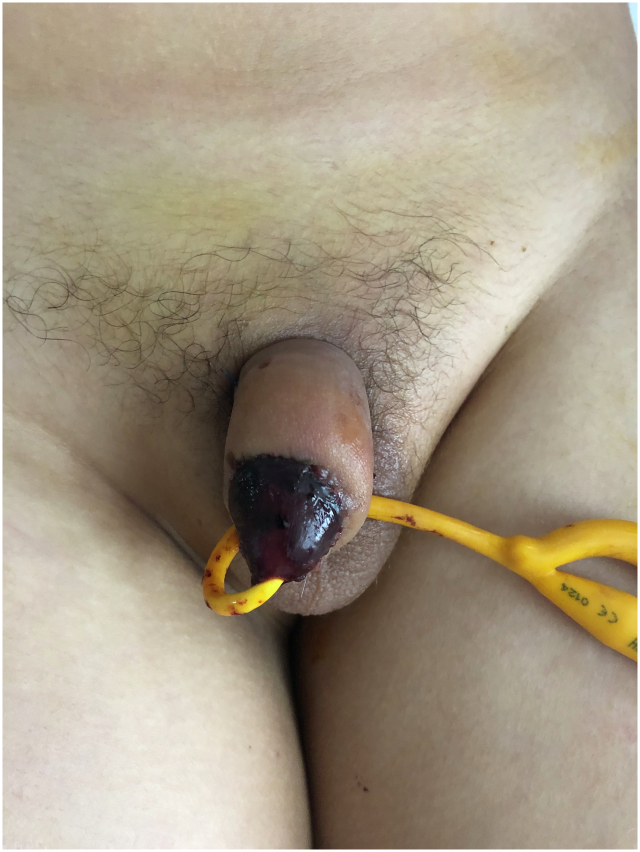
The composite glans penis graft started to turn purple on the 3rd day after surgery. (For interpretation of the references to color in this figure legend, the reader is referred to the web version of this article.)

**Fig. 5:**
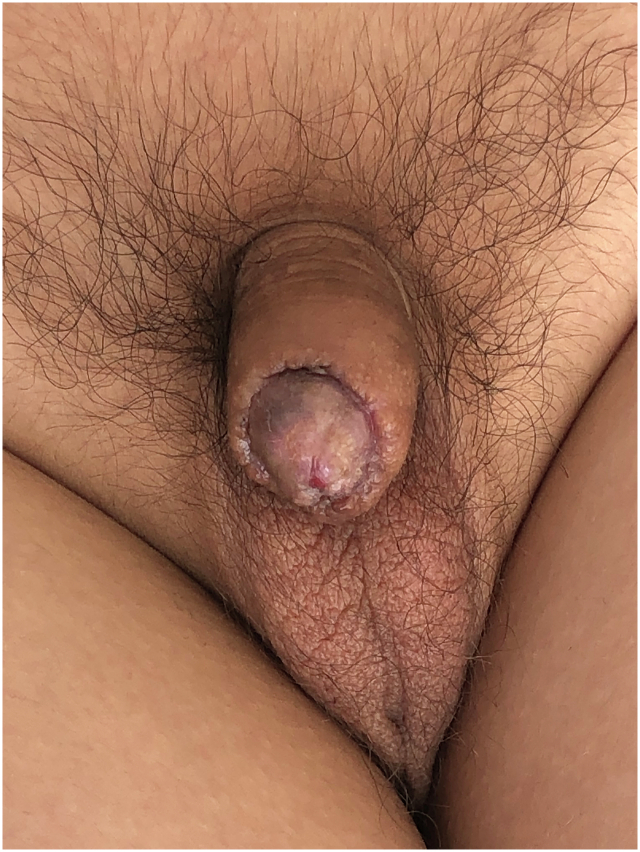
View of the glans penis at 1 year postoperatively.

A 12-year-old patient presented with glans penis amputation following circumcision. The amputated segment experienced a cold ischemia period of approximately 6 h (Fig. 1). The amputated part was wrapped in saline-moistened gauze, placed in a nylon bag, and transported in a box with ice packs. The urethra was repaired by passing a Foley catheter through the glans penis and the amputation stump as the first step (Fig. 2, Fig. 3). The proximal and distal cut ends of the corpora were then aligned, and the surrounding tunica albuginea and the septum were repaired. The composite glans penis graft and amputation stump were injected with approximately 3 ml of papaverine solution with a 22-gauge needle every 4 h. The composite glans penis graft was kept constantly moist with chlorhexidine gauze dressing and oxytetracycline-based fatty antibiotic ointment. Increased local blood leakage was observed from wound edges, but a blood transfusion was not needed for the patient. The composite glans penis graft started to turn purple on the 3rd day after surgery. The venous bleeding was observed using a pinprick on the graft on the 7th day after surgery (Fig. 4). The color of the glans penis began to lighten from the 15th day onwards and returned to its standard skin color by 1 month postoperatively (Fig. 5). Sensation over the glans was present at 2 years postoperatively. This may be explained by spontaneous reinnervation, a phenomenon also reported in the free flap literature.

## 3 Discussion

Penile replantation is one of the least frequently reported microsurgical procedures in the literature [4]. Non-circumcision-related penile amputations typically occur at the proximal level, where vessel diameters are sufficient for microsurgery. Furthermore, successful replantation of a second self-amputated penis has been reported in the literature due to available vessel diameters. In contrast, circumcision-related amputations typically involve the distal level, usually affecting the glans penis, making microsurgical repair challenging.

Several surgical methods for penis reconstruction have been described, although there is no cure for partial penis loss. Reimplantation of the glans penis is very important to avoid shortening and aesthetic loss. To date, only a single case of glans penis replantation has been reported in the literature [5]. Glans penis amputations are mentioned in a few review articles describing complications after circumcision, but there is no information on treatment [6,7]. Only the use of 2 composite glans penis graft has been reported for distal-level penis amputations [7,8]. Glans penis reimplantation without repair of the dorsal vessels of the penis could be considered as a composite graft. Thus, the composite graft will survive by imbibition from the penis stump [9]. The glans penis composite graft is successful because the dorsal and urethral arteries serve as an excellent source of blood supply to the corpus spongiosum and the glans. Blood circulation is easily established through the spongy tissue of the grafted penis [10]. Furthermore, the corporal sinusoidal blood flow could also act as a diffusion for the composite graft.

Papaverine inhibits phosphodiesterase isoenzyme 3, which is present in the cytosol of smooth muscle cells. This inhibition prevents the inactivation of secondary messengers, including cyclic adenosine monophosphate (cAMP) and cyclic guanosine monophosphate (cGMP). The accumulation of these second messengers results in a decrease in intracellular calcium levels, leading to smooth muscle relaxation and vasodilation [11]. The experimental study found higher CD34 and VEGF levels with the use of prolonged-release papaverine, demonstrating increased angiogenesis and an anti-inflammatory effect [12]. We applied a continuous papaverine solution in the composite graft and penile stump, and the injection frequency was planned depending on the half-life of the drug. Papaverin diffusion is facilitated by the spongy tissues. Papaverin diffusion is easier due to the penis's spongy tissues, and this texture provides a continuous environment for exposure to the drug.

Local application both increases the level of effect and protects the patient from systemic side effects [13,14]. Like any graft fed by imbibition, the composite glans penis graft turns purple within a few days after surgery. However, with the development of neogenesis, the color of the glans penis lightens and initially becomes pink, then returns to its standard color. The use of papaverine to enhance graft neogenesis has not been previously reported.

## 4 Conclusion

We believe that topical papaverine will increase neovascularization and thus minimize graft loss. This effect may be particularly valuable in thick tissues such as composite grafts, where survival is often more challenging.

## Author contribution

The case presented in this article, as well as all stages of its documentation and manuscript preperation,were entirely conducted by the author.

## Consent

Written informed consent was obtained from the patient's parents/legal guardian for publication and any accompanying images. A copy of the written consent is available for review by the Editor-in-Chief of this journal on request.

## Ethical approval

This is urgent surgery and we have no chance to take ehical approval. I mean this procedure does not need for this case. My instution is Hacettepe Universty, Ankara, Türkiye.

## Guarantor

Serdar Nasır MD.

## Research registration number

Not applicable.

## Funding

I do not have any funding support.

## Conflict of interest statement

I declare that I have no conflicts of interest.
